# Fracture Behavior and Mechanism of Ceramic Fiber Insulation Tiles: An Analysis Based on Experiment and Numerical Simulation

**DOI:** 10.3390/ma19153246

**Published:** 2026-07-31

**Authors:** Yiming Wang, Hong Ye, Xiaoliang Ma, Xuefeng Mu, Kaili Yin, Meng Cui, Baojun Zhao, Yuanpeng Fang, Yesheng Zhong, Liping Shi, Xiaodong He

**Affiliations:** 1 National Key Laboratory of Science and Technology on Advanced Composites in Special Environments, Harbin Institute of Technology, Harbin 150080, China; 21b918026@stu.hit.edu.cn (Y.W.); shiliping@hit.edu.cn (L.S.);; 2 Chengdu Aircraft Design and Research Institute, Aviation Industry Corporation of China, Chengdu 610091, China

**Keywords:** ceramic fiber insulation tile, three-dimensional network model, finite element (FE) model, crack size, fracture behavior

## Abstract

Ceramic fiber insulation tile (CFIT) is a brittle porous ceramic fiber material. Its three-dimensional random network structure yields low density, high porosity, low thermal conductivity, and excellent high-temperature stability, making it a widely used material in aerospace thermal protection systems. This renders it particularly crucial to explore fracture dimensions, improve the fracture toughness of materials, and circumvent internal defects. In this study, the numerical simulation and experimental approaches are employed to investigate the fracture behavior and toughening mechanism of CFIT. First, a three-dimensional network model and macroscopic finite element (FE) model of CFIT are established, and the validity of the macroscopic FE model is confirmed by comparison with fracture experimental results. Meanwhile, the effects of CFIT porosity, fiber length, fiber diameter, and fiber orientation angle on fracture toughness are systematically investigated. Furthermore, based on practical requirements, the dimensions and structure of the ceramic fibers are determined, thereby elucidating the mechanism through which changes in crack size influence fracture behavior. Finally, fracture behavior under the macroscopic model is analyzed by introducing different crack sizes and prefabricated defects, and the influence laws of crack size and prefabricated defects on CFIT are determined. In summary, this study provides theoretical guidance for research on the fracture behavior of porous ceramic fiber materials.

## 1. Introduction

Fiber porous materials, such as bone, wood, paper and ceramic, are characterized by a three-dimensional random network structure and widely present in both natural and engineering systems [1,2,3]. The mechanical response of their internal defects significantly diverges from that in continuous media [4,5,6,7]. Ceramic fiber insulation tile (CFIT), a type of brittle ceramic fiber porous material, has been successfully implemented in thermal protection systems for aerospace vehicles owing to its high porosity, low density, low thermal conductivity [8], and superior high-temperature stability, but its inherently brittleness also makes it highly sensitive to tensile loads during service [9,10,11]. And its deformation and failure mechanisms are notably complex, often encompassing multiple coupled processes, such as large-strain deformation, fiber orientation, porosity, and fiber fracture [12,13,14]. 

The CFIT’s fracture property is not solely governed by the raw material property, but is more critically determined by the geometric attributes of their internal microstructure [15]. The geometry and spatial arrangement of these microstructural features can significantly modulate the material’s macroscopic fracture behavior [16,17]. Research approaches for the fracture behavior of porous materials primarily include numerical modeling, experimental analysis, finite element (FE) method, and extended finite element method (XFEM) simulations. In earlier studies, Zheng et al. [18] developed a numerical model for predicting the fracture property of porous materials and systematically validated it through three-point bending tests on specimens with different porosities. Cramer [19] experimentally demonstrated that the spatial arrangement of pores critically influences the fracture toughness of aluminum plates containing multiple circular holes. Keles et al. [20,21] employed the FE method integrated with classical fracture mechanics theory to study the fracture behavior of porous materials containing circular pores under axial and biaxial loading. They systematically analyzed the fracture characteristics of brittle porous materials with random crack orientations. Rezanezhad et al. [22,23] employed XFEM to simulate dynamic crack propagation in porous materials, revealing crack–pore interaction mechanisms and systematically analyzing how crack paths are influenced by pore orientation and location.

Furthermore, research indicates that ceramic fibrous porous materials demonstrate a notable resistance to brittle fracture characteristics [24,25]. Chen and Isaksson systematically investigated the fracture behavior of porous materials with different densities using the FE method, determining the ratio of initial crack length to pore diameter corresponding to crack-insensitive behavior [26,27]. Zhang et al. [28,29] developed a three-dimensional numerical model integrated with FE analysis to study fracture in fibrous network materials. The results show that crack insensitivity below critical length stem from toughening via damage accumulation near the crack tip and fiber rearrangement. Additionally, crack propagation perpendicular to its path is effectively suppressed, thereby increasing the material’s load-bearing capacity. Li [30,31] investigated the dynamic fracture of fibrous porous materials via Split Hopkinson Pressure Bar (SHPB) test, revealing pronounced loading-rate sensitivity in fracture properties. The developed XFEM model effectively simulates both static and dynamic fracture, revealing the influences of temperature and loading rate on material fracture toughness. It provides a valuable reference for evaluating fracture toughness under extreme environmental conditions [32,33]. Consequently, the investigation of fracture behavior is constrained by service conditions and the existing mesoscopic model scale, and remains deficient in studies concerning both the effects of mesoscopic parameters on fracture toughness and the role of prefabricated defects in macroscopic fracture behavior. 

In this paper, a three-dimensional network model and a macroscopic FE model of CFIT are developed, and the validity of the macroscopic model is confirmed by comparison with fracture experiments. The effects of key microstructural parameters—including porosity, fiber length, fiber diameter, and fiber orientation angle—on fracture toughness are systematically examined. On this basis, taking practical requirements into account, the dimensions and structure of the ceramic fibers are optimized, and the mechanism by which crack size variation affects fracture toughness is further elucidated. Moreover, macroscopic fracture behavior is investigated by introducing pre-existing defects and different crack sizes into the model.

## 2. Material and Experiment

### 2.1. Material

**Figure 1:**
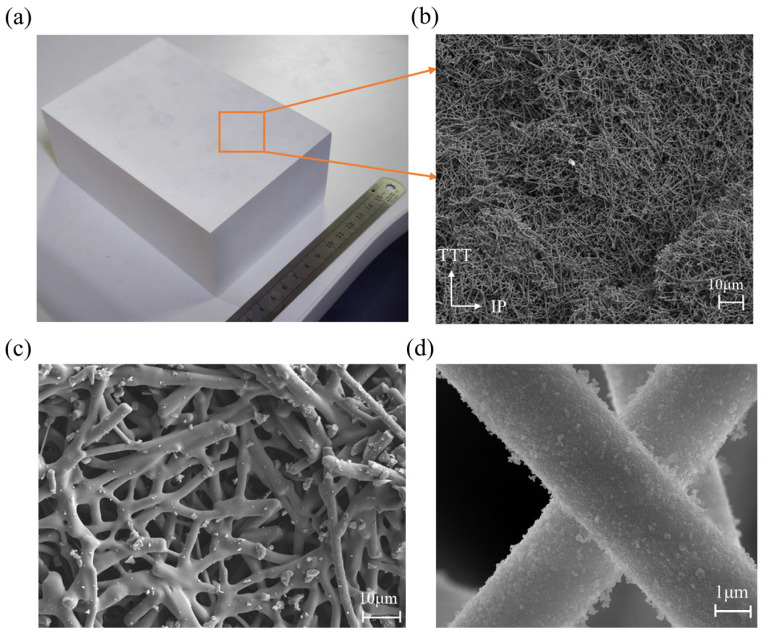
Ceramic fiber insulation tile: (**a**) photograph; (**b**–**d**) SEM image showing microstructure.

CFITs are fabricated by controlling the fiber aspect ratio and tailoring the filler composition through a sequence of processing steps that include dispersion, suction filtration, drying, and high-temperature sintering. Further details regarding the specific procedures and parameters of the preparation process can be found in Ref. [34]. The resulting material possesses a 3D random network structure, which endows it with outstanding high-temperature dimensional stability. Figure 1 presents a CFIT sample and its corresponding scanning electron microscopy (SEM) photograph. The SEM used in this study was a TESCAN AMBER model (TESCAN, Brno, Czech Republic). The accelerating voltage, working distance, and detector type were 20 kV, 8 mm, and a secondary electron detector, respectively. Sample preparation was carried out as follows: a thin slice of about 2–3 mm thickness was first cut from the cross-section of the specimen in the thickness direction, then attached to the sample stub using conductive adhesive. Since the specimen itself is non-conductive, a gold sputter coating was applied for 300 s. As a typical three-dimensional random network material, the CFIT comprises numerous randomly interwoven ceramic fibers, forming a highly porous and non-oriented structure. The SEM image clearly shows that these fibers interconnect through mutual overlapping, creating a stable and complex spatial network. This unique structure provides excellent thermal insulation and mechanical property, along with transverse isotropy.

### 2.2. Fracture Experiment

**Figure 2:**
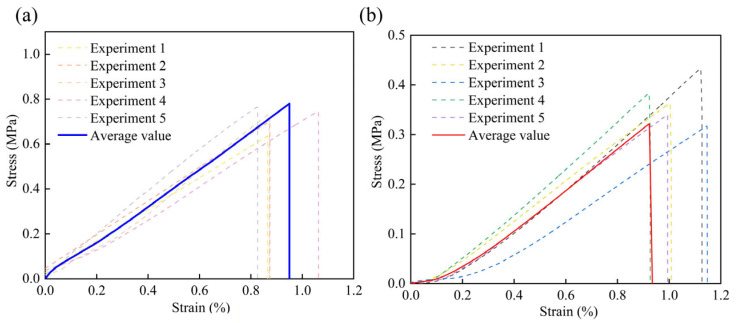
Experimental result of CFIT under tensile loading: (**a**) intact sample; (**b**) pre-notched sample.

**Figure 3:**
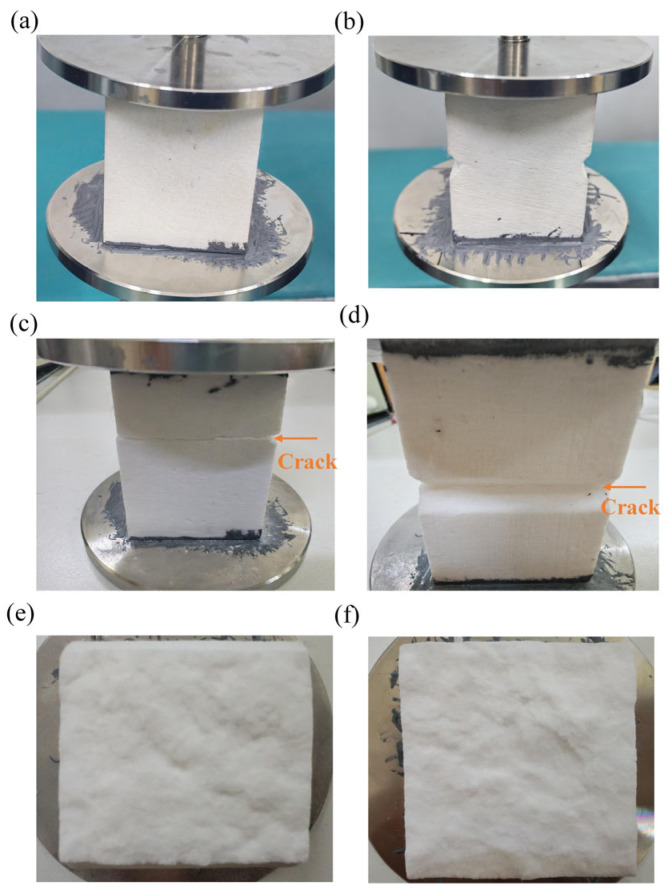
Ceramic fiber insulation tile fracture experiment comparison: (**a**) intact pre-test; (**b**) pre-notched pre-test; (**c**) intact post-test; (**d**) pre-notched post-test; (**e**) intact cross section; (**f**) pre-notched cross section.

According to the ASTM E399 standard [35], a tensile fracture specimen was designed with overall dimensions of 40 mm × 40 mm × 50 mm. The crack size is defined by the ratio of crack length to thickness, with a value of 0.2. To assess the reproducibility of the experimental data, fracture tests were carried out under tensile loading on both intact and pre-cracked specimens, with five replicates per condition; the obtained results are depicted in Figure 2. As shown in Figure 2, both the intact specimens and the prefabricated fracture specimens exhibited brittle fracture. Based on five repeated experiments, the average tensile strengths of the intact specimens and the fractured specimens for CFIT were determined to be 0.78 MPa and 0.32 MPa, respectively. The port section retains widths of 4 mm on both sides, as detailed in Figure 3. For intact specimens, fracture frequently initiates in the mid-to-upper region (Figure 3c). However, the significant stress concentration at the crack tip drives fracture to invariably originate at the defect site in specimens containing pre-existing cracks (Figure 3d). Marked differences exist between the fracture cross-sections: the intact specimen presents a rough and undulating surface (Figure 3e), indicative of a tortuous crack path and effective energy dissipation. Conversely, the pre-cracked specimen displays a uniform and smooth morphology (Figure 3f), characteristic of rapid crack advancement along the predefined path and intense local stress concentration.

**Figure 4:**
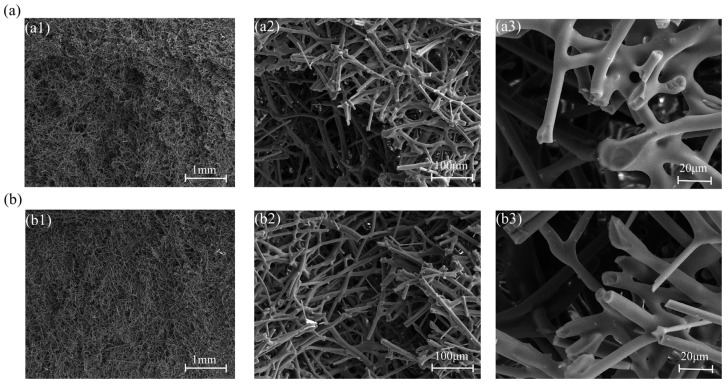
SEM results of the CFIT’s fracture surface: (**a1**–**a3**) intact specimen; (**b1**–**b3**) pre-notched specimen.

Figure 4 shows the SEM observation results of the CFIT’s fracture surface. Significant differences in fiber fracture characteristics under tension exist between intact and pre-notched specimens. In the intact specimen, energy dissipation occurs primarily through microcrack deflection and brittle fiber fracture. Fiber fracture is typically delayed, and the fracture surface exhibits a distinct layered morphology. This results from the crack path preferentially deviating around high-strength regions, leading to successive delamination fracture under tension force. In contrast, the pre-notched specimen features a predetermined crack propagation path, resulting in a fracture surface lacking layered structures. Constrained to a narrow crack path, the energy release rate rises significantly, causing pronounced localization of fiber fracture at the crack tip. The fracture surface shows sharp morphologies characteristic of typical brittle fracture. These differences fundamentally stem from the stress concentration severity and crack propagation behavior. In the intact specimen, the absence of a pre-existing crack allows stress to disperse along multiple paths, promoting fiber fracture primarily by shear or torsion. In the pre-notched specimen, however, strong stress concentration at the crack tip forces fibers to fail by direct tensile tearing, resulting in a sharp, regular, and distinctly brittle fracture morphology.

## 3. Simulation Method and Model Validation

### 3.1. Simulation Method

To simulate material damage in a network structure subjected to tensile loading, the stress distribution was calculated across individual elements throughout the loading procedure using numerical analysis methods. Strength and stability criteria are employed to assess element failure [36,37]. For strength-related failure, the maximum stress criterion was adopted, whereas for stability-related failure, the column stability criterion was applied [38]. To construct a double-edge crack model, the coordinate of the intersection point between the crack plane and the fiber must first be determined. If the coordinates of a single fiber are given as (*x*_1_, *y*_1_, *z*_1_) and (*x*_2_, *y*_2_, *z*_2_), then the parametric equations of the line containing this fiber can be expressed as follows:

(1) $$\;x\;=\;x_{1}\;+\;k(x_{2}-x_{1})\;\;y\;=\;y_{1}\;+\;k(y_{2}-y_{1})\;\;z\;=\;z_{1}\;+\;k(z_{2}-z_{1})$$

Meanwhile, the plane on which the crack initiates is defined by:

(2) $$Z\;=\;\frac{l_{z}}{2}$$

By combining Equations (1) and (2), the coordinates of the intersection point ($x_{\mathrm{intersect}}$, $y_{\mathrm{intersect}}$, $z_{\mathrm{intersect}}$) between the fiber and the plane can be derived as follows:

(3) $$x_{\mathrm{intersect}}\;=\;x_{1}\;+\;(\frac{l_{z}}{2}-z_{1})(x_{2}-x_{1})/(z_{2}-z_{1})\;\;y_{\mathrm{intersect}}\;=\;y_{1}\;+\;(\frac{l_{z}}{2}-z_{1})(y_{2}-y_{1})/(z_{2}-z_{1})\;\;z_{\mathrm{intersect}\;}=\;\frac{l_{z}}{2}$$

where k is the slope of the line equation representing the fiber, with a value in the range (0, 1), and $l_{z}$ is the model height in the Z-direction.

**Figure 5:**
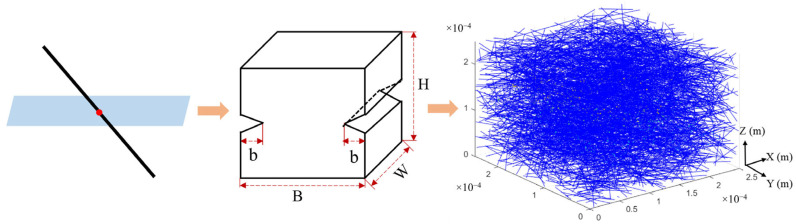
Meso-mechanical model of CFIT. (Where B represents the model width; W is the model thickness; H is the model height; *b* is the single-side crack length).

The meso-mechanical model is established as shown in Figure 5. The simulated sample is *l_x_* × *l_y_* × *l_z_* (0.25 mm × 0.25 mm × 0.25 mm). The fibers in the sample are quartz fibers, with their elastic modulus, shear modulus, fracture strength, and density being 78 GPa, 33.3 GPa, 5 GPa, and 2.2 g/cm^3^, respectively. The fiber percent is 10~20%, the fiber length is L (0.05–0.15 mm), and the fiber diameter is D (2.5~3.5 μm). Non-periodic boundary conditions were applied to the meso-mechanical model. In the model, Mode-I cracks of varying lengths were introduced. The load displacement and zero displacement were assigned to the upper, while the lower boundaries of the model, respectively. The other four surfaces were free boundaries. Numerical simulations were conducted to obtain node displacements, which were used to analyze the stress–strain distribution characteristics within the cracked model under tensile loading.

**Figure 6:**
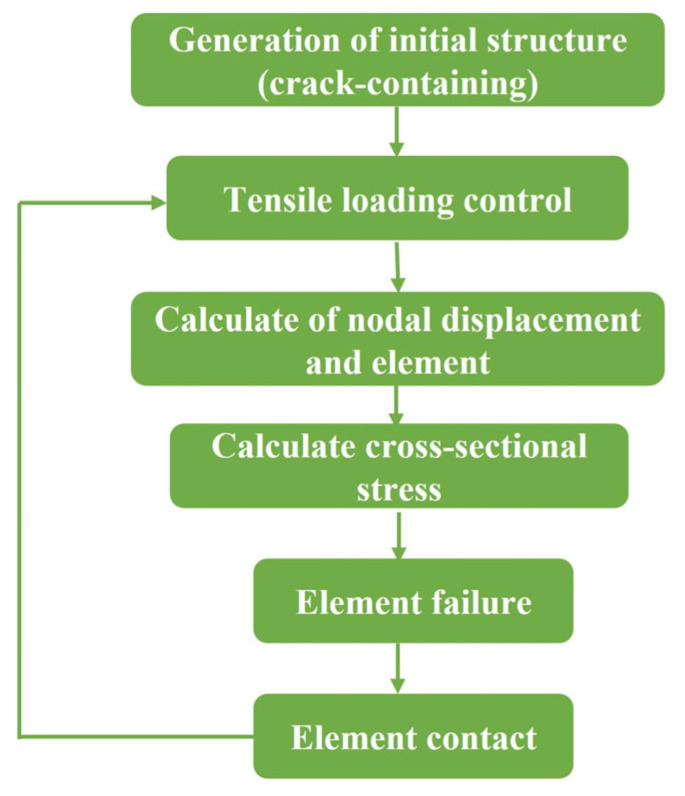
Flow chart for simulation calculation.

Based on the intersection points, the midpoint coordinates of each fiber were obtained, respectively. Then, according to the fundamental principles described in Section 3.1, the stress–strain relationship of the cracked model is calculated. Figure 6 is the flow chart for simulation calculation. First, a random fiber network model was established, and tensile loading was applied. Through the calculation of each node’s displacement and load, nodes associated with failed fibers are removed, and equivalent stiffness is assigned to elements satisfying the contact criteria. Subsequently, the displacement of the deformation layer is incrementally increased until it reaches a predefined value. Finally, repeat the above cycle until the calculation ends.

**Figure 7:**
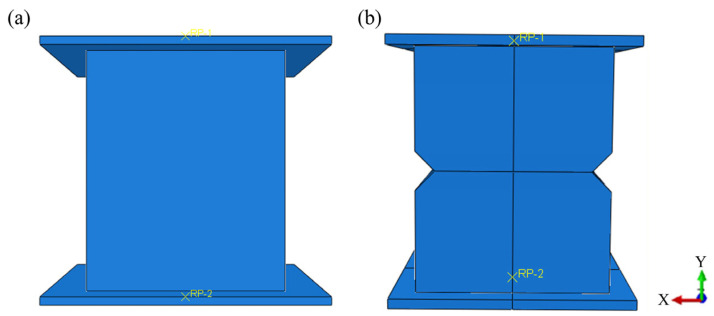
The FE model of CFIT: (**a**) intact CFIT; (**b**) pre-notched CFIT.

Based on four fundamental assumptions, and within the elastoplasticity theoretical frameworks [39,40], a macroscopic constitutive model suitable for CFIT is developed by introducing a progressive damage method to modifying the Hashin yield criterion. This model is designed to systematically investigate the macroscopic fracture behavior of the CFIT under tensile loading. Figure 7 presents the FE model of the CFIT with prefabricated fracture, where the fracture size is adjustable to meet the demands of specific simulation analyses. The boundary condition was defined as a fixed constraint on the bottom surface, and the mesh element type was set to C3D8. The specific material parameters, theoretical approaches, and numerical implementation details employed in the model construction are all drawn from and supported by Ref. [34].

In accordance with ASTM E399 [35], the fracture toughness of CFIT can be quantitatively determined by implementing the calculations specified in Equations (4)–(6).

(4) $$K_{\mathrm{IC}}=(P/TW^{1/2})f\left(2b/W\right)$$

(5) $$T={{BB}_{N}}^{1/2}\;$$

(6) $$f(\frac{2b}{W})=\frac{(2+\frac{2b}{W})(0.886+4.64\frac{2b}{W}\;-\;13.32{\left(\frac{2b}{W}\right)}^{2}+14.72{\left(\frac{2b}{W}\right)}^{3}-\;5.6{\left(\frac{2b}{W}\right)}^{4})}{{(1\;-\;\frac{2b}{W})}^{3/2}}$$

where P is the load during the loading process. In this paper, the values of *P* are taken as $P_{\max}$ (the maximum load) and $P_{Q}$ (the load at the 95% position). $B_{N}$ is the width at the crack tip.

### 3.2. Model Validation

**Figure 8:**
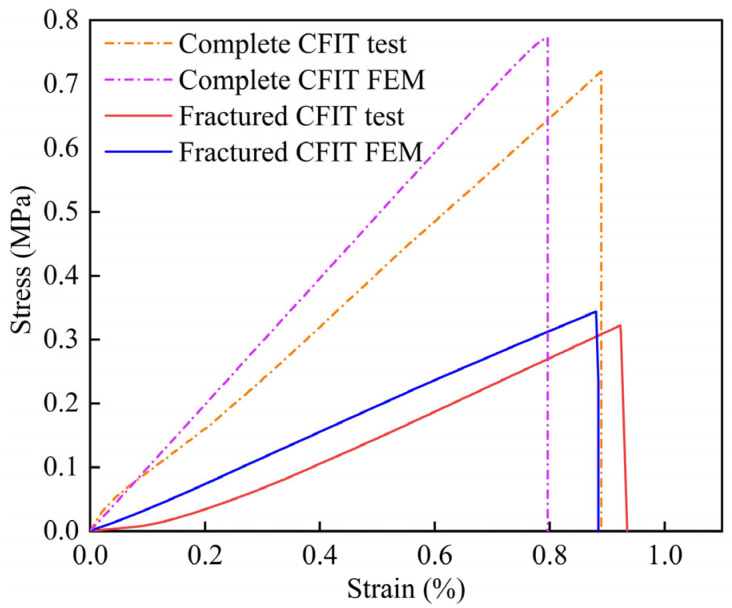
Experimental and numerical mechanical response curves of CFITs under tensile loading.

A fixed boundary condition was applied to the bottom surface, and the element type was set to C3D8. All mesh sizes are given in millimeters (mm). To determine the appropriate mesh size in FE simulation, four mesh refinement strategies were devised for the CFIT. The corresponding mesh region division and convergence result are presented in Supplementary Materials Table S1 and Figure S1, respectively. Based on the convergence result, the non-fracture region was assigned a mesh size of 2.0, while the fracture region was locally refined to 1.414. Figure 8 shows the tensile response curve of the macroscopic model under tensile loading. Correspondingly, the macroscopic model predicted tensile strengths of 0.75 MPa for the intact CFIT and 0.35 MPa for the notched CFIT. The tensile strengths calculated for the intact and pre-notched CFITs deviate from the experimental values by 3.8% and 9.4%, respectively. These experimental and numerical results demonstrate that the presence of a prefabricated notch leads to an approximate 54% reduction in tensile strength. The results above illustrate that the constitutive model can effectively predict the macroscopic fracture behavior of CFITs under tensile loading.

## 4. Results

### 4.1. Mesoscopic Model

#### 4.1.1. Porosity

**Figure 9:**
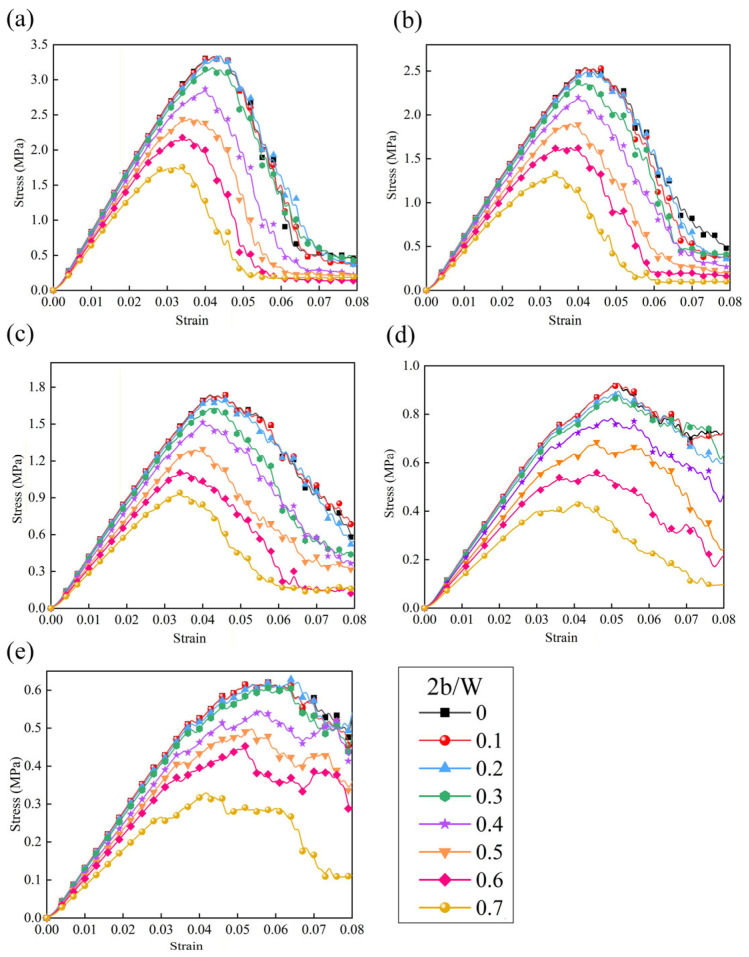
Stress–strain curves with different 2b/W and different porosities: (**a**) Porosity = 82%. (**b**) Porosity = 84%. (**c**) Porosity = 86%. (**d**) Porosity = 88%. (**e**) Porosity = 90%.

To reduce the confounding influence of parametric coupling on our conclusions, this study adopted a controlled approach by investigating each parameter independently: all non-target parameters were kept near baseline, while target variation was confined to feasible manufacturing ranges. These measures were intended to minimize property shifts arising from unintended coupling effects. Figure 9 illustrates the stress–strain curves of CFIT under different crack sizes and porosities. The cracked material model stiffness is derived from the slope of the stress–strain curve’s initial linear stage. Additionally, porosity demonstrates a negative correlation with material strength and stiffness, meaning that higher porosity corresponds to lower strength and stiffness. As shown from Figure 9a–c, when the crack size (crack size-to-thickness ratio 2b/W) ranges from 0.1 to 0.2, the stress–strain curves of low-porosity models (porosity = 82~86%) exhibit near-complete overlap. In contrast, the stress–strain curves of the high-porosity models (porosity = 88%, 90%) exhibit near-complete overlap when 2b/W is ≤0.3, as depicted in Figure 9d,e. The findings demonstrate that microcracks cannot significantly affect the mechanical behavior of CFIT under these conditions. Notably, all curves exhibit typical brittle fracture characteristics after reaching the peak stress, which is highly consistent with the micro-damage mechanism of crack propagation within CFIT.

**Figure 10:**
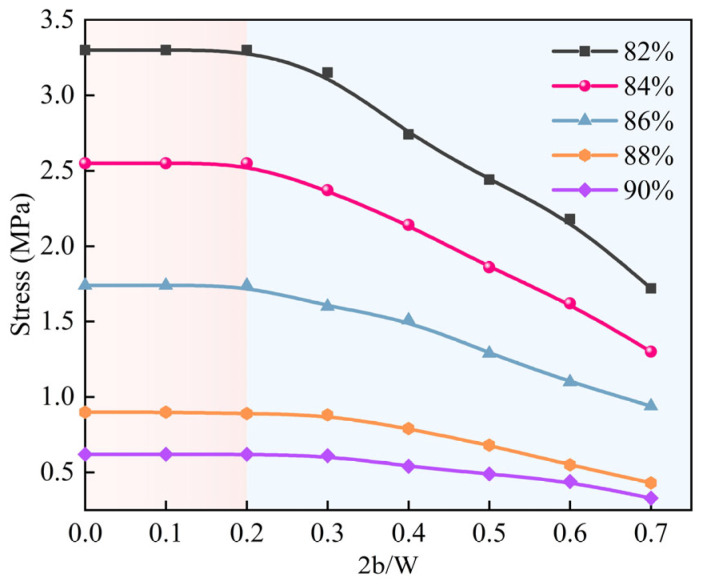
The maximum stress with different 2b/W and different porosity.

The calculation results in Figure 10 illuminate the variation in maximum stress with the ratio of 2b/W. When porosity is below 88%, 2b/W stabilizes at 0.2, suggesting that cracks have a negligible effect on the maximum stress, as shown in Figure 10. As porosity increases, the 2b/W ratio of CFIT rises to 0.3, indicating significantly enhanced crack-insensitive behavior and a larger critical crack length in high-porosity models. Moreover, when 2b/W exceeds 0.4, the maximum stress of all cracked models begins to decline sharply, demonstrating that stress concentration significantly compromises the material’s load-bearing capacity.

**Figure 11:**
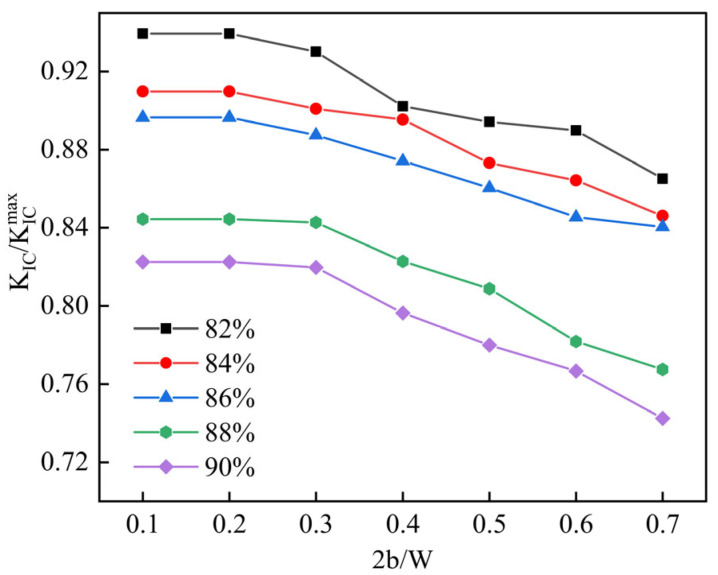
Fracture toughness with different 2b/W and different porosity.

Due to the small size of the model, the fracture toughness calculated by the method specified in the standard cannot quantitatively characterize the true fracture toughness of CFIT. Consequently, the ratio of $K_{\mathrm{IC}}$ values at the maximum load and 95% load is derived to characterize the material’s fracture toughness behavior. Figure 11 shows the ratio of CFIT’s $K_{\mathrm{IC}}$ and $K_{\mathrm{IC}}^{\max}$. It can be seen from Figure 11 that the fracture toughness of CFIT exhibits a decreasing trend with the increase in porosity. When the porosity is ≥88%, the reduction rate of the CFIT’s fracture toughness with respect to crack length is below 10.59%. However, for CFITs with a porosity ˂88%, the reduction rate of their fracture toughness with respect to crack length is less than 6.96%.

#### 4.1.2. Fiber Length

**Figure 12:**
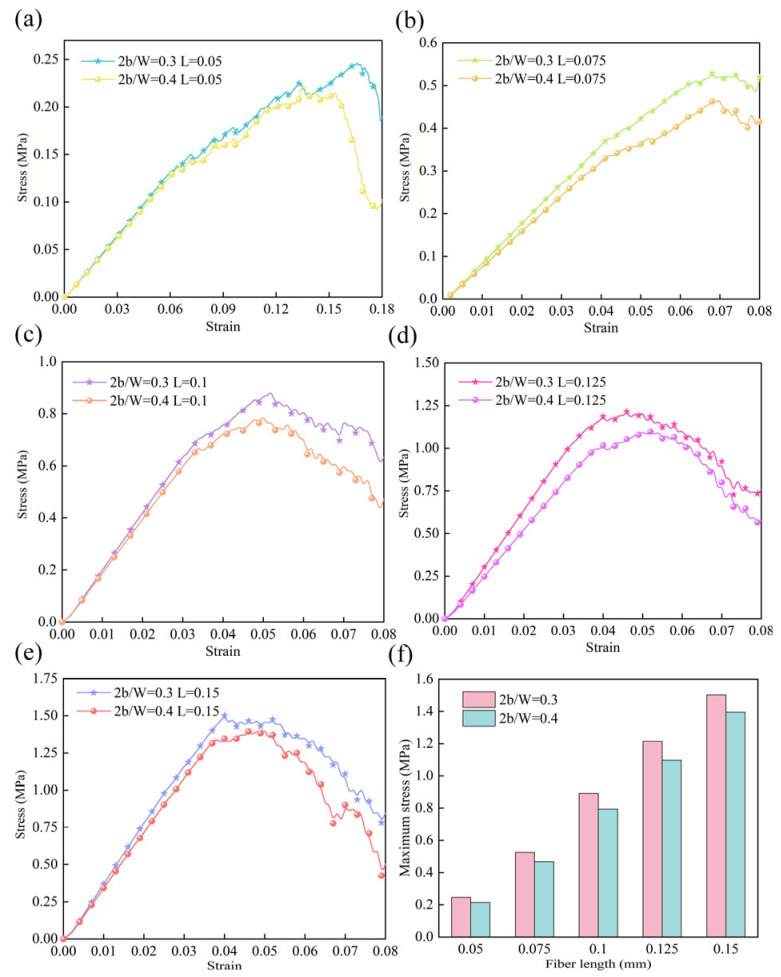
Stress calculation results with different 2b/W and fiber length: (**a**) L = 0.05 mm; (**b**) L = 0.075 mm; (**c**) L = 0.1 mm; (**d**) L = 0.125 mm; (**e**) L = 0.15 mm; (**f**) maximum stress.

Figure 12 shows the stress calculation results with different 2b/W and fiber length. As shown in Figure 12, the fiber length exhibits a positive correlation with the maximum stress by CFIT. The maximum stress range of CFIT is 0.2~1.5 MPa.

**Figure 13:**
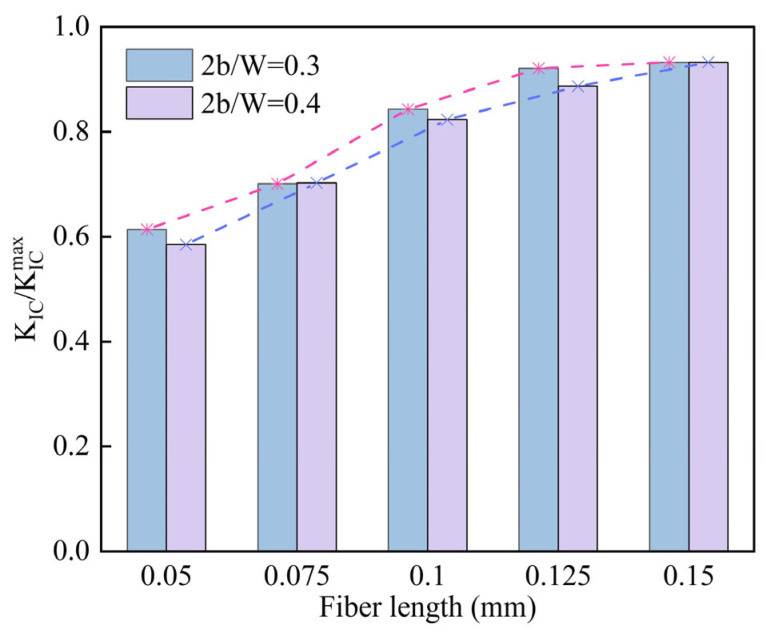
Fracture toughness with different 2b/W and fiber length.

Figure 13 illustrates fracture toughness with different 2b/W and fiber length. It can be seen from Figure 13 that a certain improvement in the CFIT’s toughness is observed when its crack size is 0.4 and the fiber lengths are 0.1~0.15 mm. Whereas, when its crack size is 0.4 and the fiber length is 0.125 mm, fracture toughness has a maximum value. In addition, the CFIT’s toughness is independent of the fiber crack size for fiber lengths of 0.075 mm and 0.15 mm.

#### 4.1.3. Fiber Diameter

**Figure 14:**
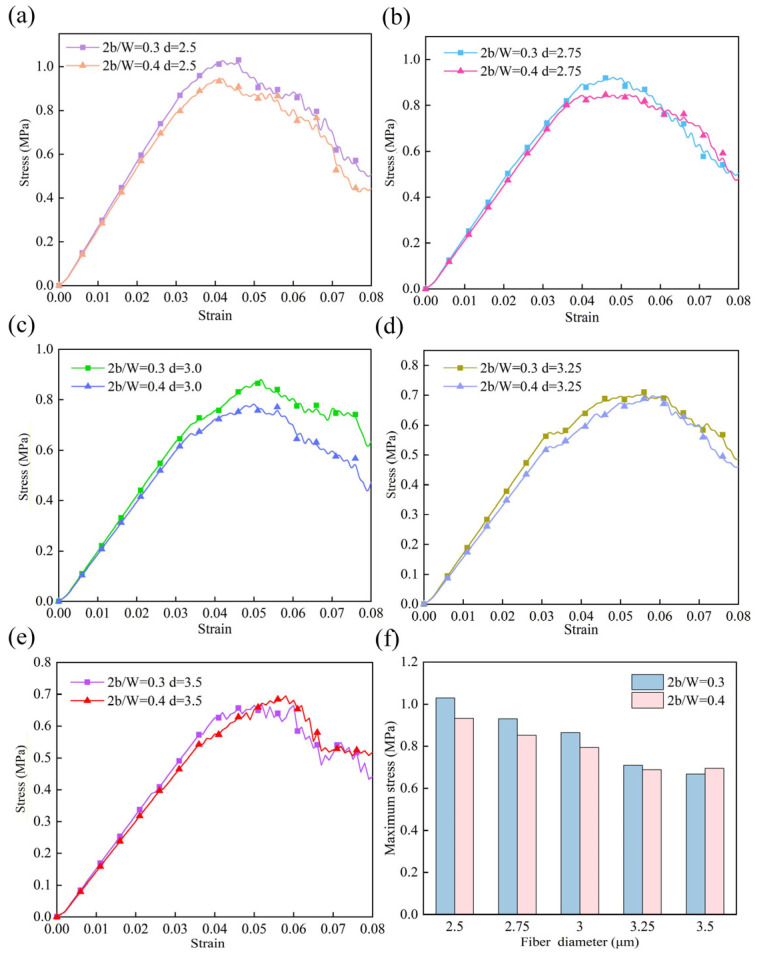
Stress calculation results with different 2b/W and fiber diameter: (**a**) d = 2.5 μm; (**b**) d = 2.75 μm; (**c**) d = 3.0 μm; (**d**) d = 3.25 μm; (**e**) d = 3.5 μm; (**f**) maximum stress.

**Figure 15:**
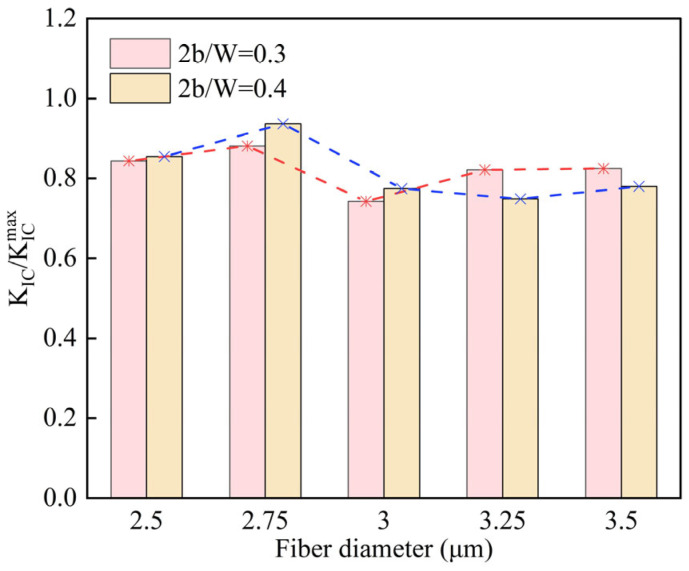
Fracture toughness with different 2b/W and fiber diameter.

Figure 14 shows the stress calculation results of different 2b/W and fiber diameter. It can be seen from Figure 14 that there is a negative correlation between fiber diameter and maximum stress. For different fiber diameters, the CFIT’s maximum stress range is 0.7~1.1 MPa. Figure 15 illustrates the fracture toughness with different 2b/W and fiber diameter. As shown in Figure 15, the CFIT’s fracture toughness exhibits a trend of first increasing and then decreasing with increasing fiber diameter. When the fiber diameter is in the range of 2.5 μm to 3 μm, the CFIT’s fracture toughness is notably influenced by the crack size. For fiber diameters of 3.25 μm and 3.5 μm, the CFIT’s fracture toughness remains essentially consistent across different crack sizes. In summary, moderately refined fibers enhance stress tolerance and fracture toughness of CFITs in the presence of cracks. Although large fiber diameters improve crack adaptability, they reduce toughening efficiency.

#### 4.1.4. Fiber Orientation Angle

**Figure 16:**
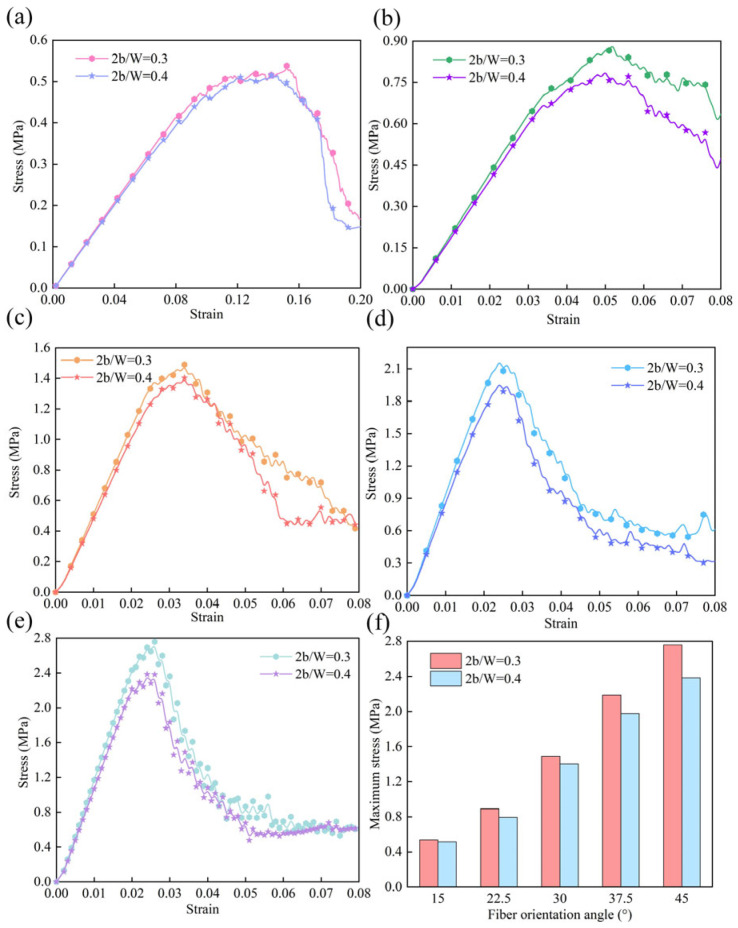
Stress calculation results of with different 2b/W and fiber orientation angle: (**a**) 15°; (**b**) 22.5°; (**c**) 30°; (**d**) 37.5°; (**e**) 45°; (**f**) maximum stress.

**Figure 17:**
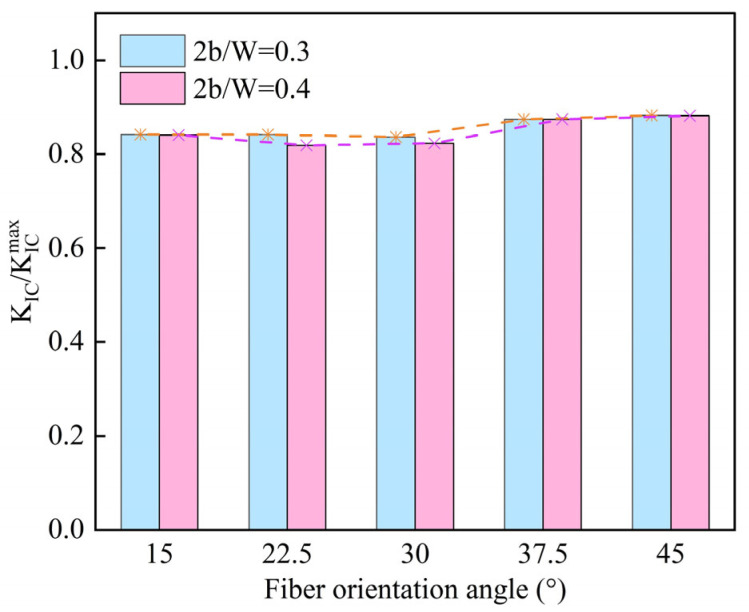
Fracture toughness of with different 2b/W and fiber orientation angle.

Figure 16 shows the stress calculation results with different 2b/W and fiber orientation. As shown in Figure 16, the fiber orientation angle is positively correlated with the maximum stress. For the fiber orientation angle, the CFIT’s maximum stress range is 0.5~2.8 MPa. Figure 17 illustrates the fracture toughness with different 2b/W and fiber orientation angle. It can be seen from Figure 17 that the fracture toughness initially decreases slowly and then levels off with increasing fiber orientation angle. At fiber orientation angles of 15°~30°, the fracture toughness remains unchanged at a crack size of 0.3, yet decreases to some extent when the crack size is increased to 0.4. In contrast, for angles of 37.5°~45°, the fracture toughness shows little variation with crack size.

#### 4.1.5. Fracture Damage

**Figure 18:**
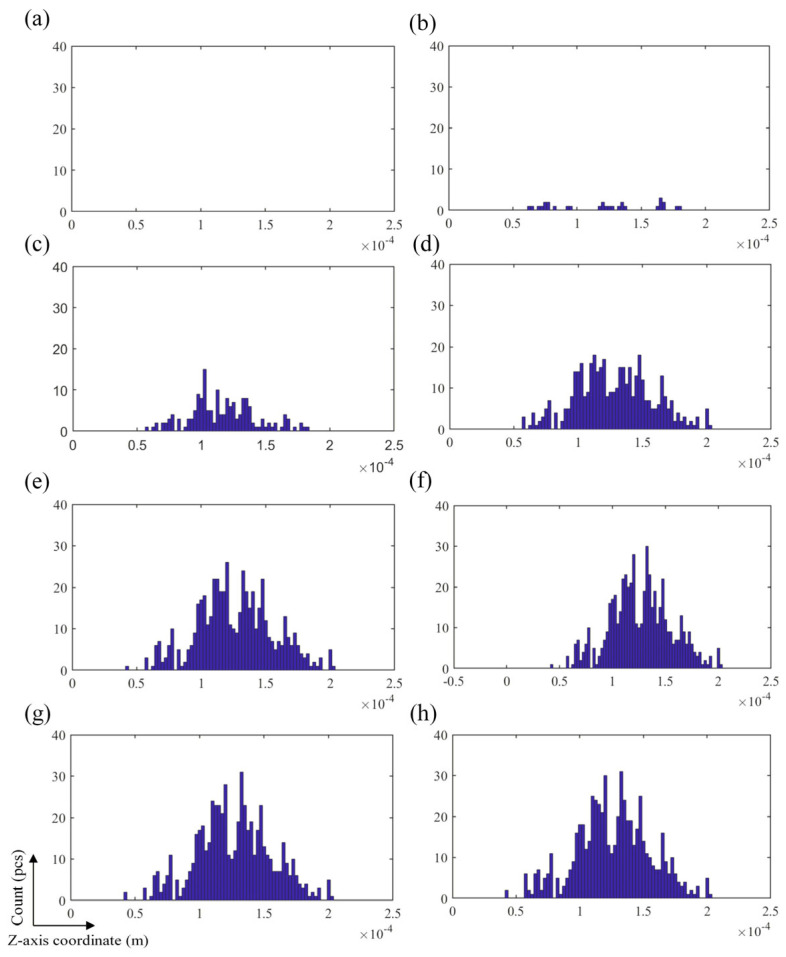
Distribution of fiber damage along the loading direction in CFIT under 2b/w = 0.3. (**a**) ε = 0.022; (**b**) ε = 0.044; (**c**) ε = 0.066; (**d**) ε = 0.088; (**e**) ε = 0.110; (**f**) ε = 0.132; (**g**) ε = 0.154; (**h**) ε = 0.176.

The CFIT and its surface coatings are required to exhibit a tensile strength of at least 0.6 MPa during service. Practical experience has demonstrated that the coating application generally reduces the tensile strength of the tiles to a certain extent. Consequently, in the spraying process, the tensile strength is typically maintained within the range of 0.8–1.0 MPa, as this range ensures more stable and reliable strength properties. This specific interval is critical for achieving optimal overall performance: compromised load-bearing capacity results from insufficient strength, while excessive strength leads to detrimental effects on the subsequent coating application process and induces through-thickness cracking upon high-temperature exposure. A CFIT with a porosity of 88%, fiber length of 0.1 mm, fiber diameter of 3 μm, and fiber orientation angle of 22.5° was selected to study fiber fracture and damage behavior during CFIT fracture. Figure 18 illustrates the evolution characteristics of fiber damage in CFIT under tensile loading, along the loading direction. Numerical simulation results indicate that the CFIT exhibits a pronounced strain-dependent mechanical response: as the strain gradually increases, the material initially displays linear elastic deformation behavior. When the strain reaches a certain critical value, internal damage begins to intensify, leading to fiber fracture. The local damage then propagates to adjacent elements, triggering contact failure and ultimately complete material fracture. The damage evolution process described above uncovers the progressive failure mechanism of CFIT, characterized by the transition from microscopic localized damage to macroscopic overall fracture. This finding provides critical insights into its tensile fracture behavior and serves as a guide for the design of material structures.

**Figure 19:**
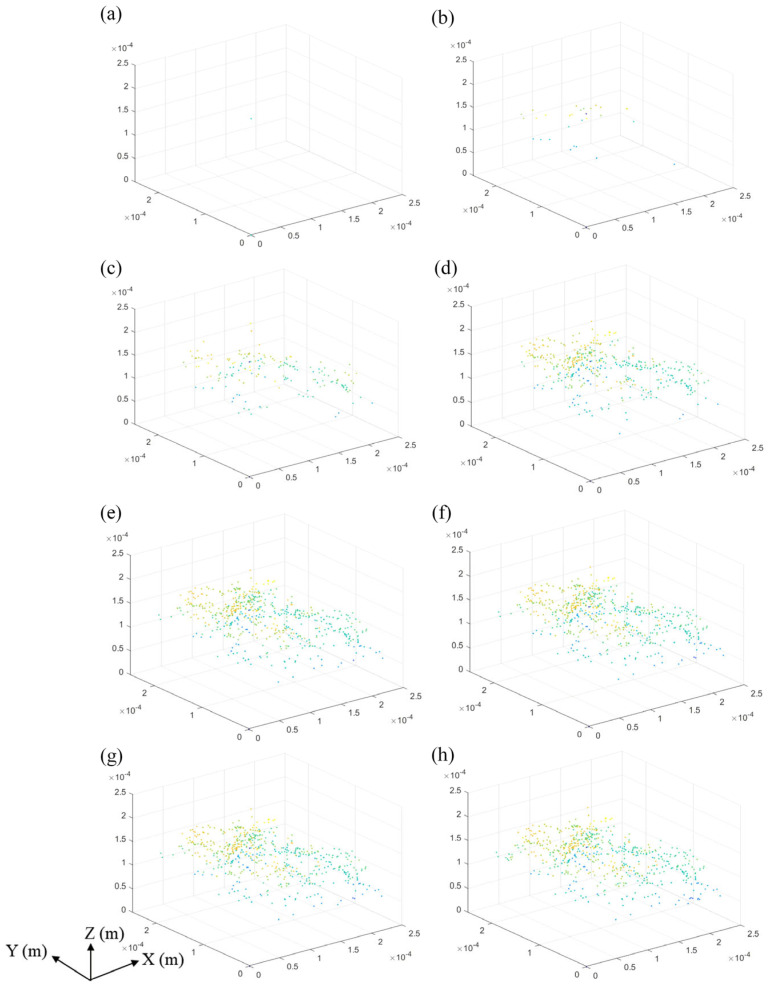
Structural damage process of CFIT along the loading direction under 2b/w = 0.3. (**a**) ε = 0.022; (**b**) ε = 0.044; (**c**) ε = 0.066; (**d**) ε = 0.088; (**e**) ε = 0.110; (**f**) ε = 0.132; (**g**) ε = 0.154; (**h**) ε = 0.176.

Figure 19 presents the damage evolution characteristics of the material subjected to tensile loading along the z-axis direction under 2b/w = 0.3. To facilitate visualization of the damage development, the z-coordinate midpoints of damaged components are color-coded, with identical colors denoting components belonging to the same horizontal layer. This color representation clearly demonstrates a layered damage propagation pattern along the specimen height: damage initially appears at z = 0.50 × 10^−4^ m, subsequently expands to z = 1.5 × 10^−4^ m and then to z = 2.3 × 10^−4^ m, and ultimately permeates the entire structure. Based on the analysis of Figure 18 and Figure 19, it can be observed that damage preferentially initiates at a specific layer near the bottom of the specimen. With increasing load, microcracks within this initially damaged layer propagate progressively, successively inducing secondary damage in adjacent layers, while the damaged zone gradually migrates toward the eventual fracture plane. Once the accumulated damage attains a critical threshold, unstable crack propagation ensues, ultimately resulting in overall brittle fracture along this damage-induced pathway. The entire failure sequence distinctly demonstrates a characteristic progressive degradation pattern. This layer-by-layer progressive fracture mode agrees well with the SEM observations in Figure 4. The SEM results show a layered structure, indicating fracture propagation along interlayer boundaries. Moreover, fiber-adjacent stress concentration is localized near the crack tip, pinpointing this region as the primary site for damage initiation and growth.

**Figure 20:**
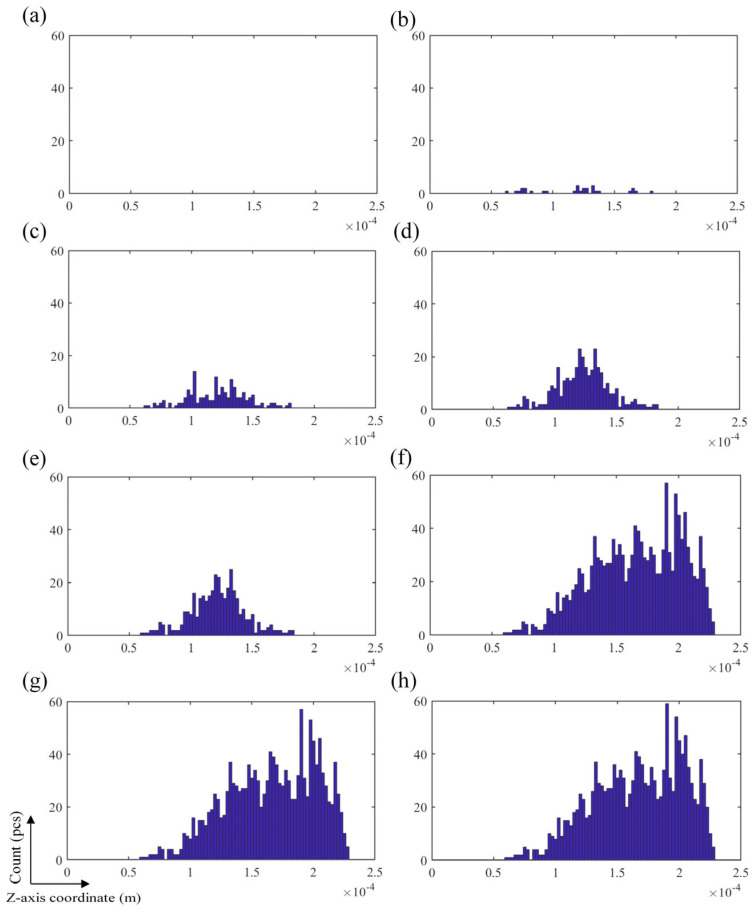
Distribution of fiber damage along the loading direction in CFIT under 2b/w = 0.4. (**a**) ε = 0.022; (**b**) ε = 0.044; (**c**) ε = 0.066; (**d**) ε = 0.088; (**e**) ε = 0.110; (**f**) ε = 0.132; (**g**) ε = 0.154; (**h**) ε = 0.176.

Figure 20 shows the distribution of fiber damage along the loading direction in CFIT under 2b/w = 0.4. As shown in Figure 20, the variation trend is generally consistent with that observed in Figure 14 (2b/W = 0.3). In both cases, linear elastic deformation occurs prior to fiber fracture, which is triggered upon reaching a critical strain value and ultimately results in complete material failure. Unlike that shown in Figure 18, as the crack size increases, the damaged elements exhibit a sharp increase within the range of z = 1.3–2.3 × 10^−4^, indicating rapid fracture of the material at this stage. This indicates that once the crack size surpasses the critical value, further widening precipitates earlier fracture.

**Figure 21:**
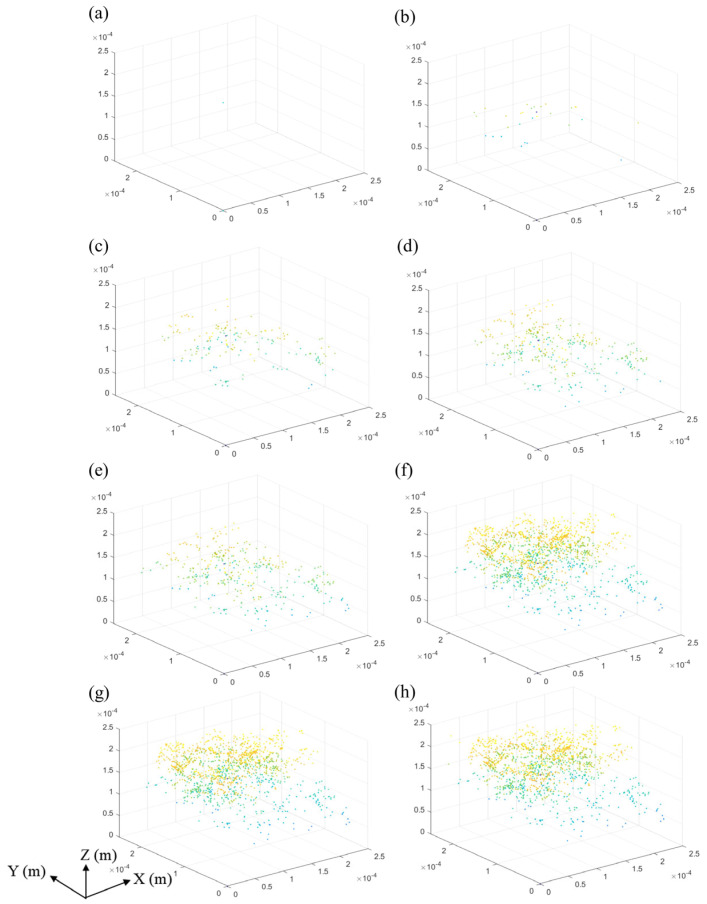
Structural damage process of CFIT along the loading direction under 2b/w = 0.4. (**a**) ε = 0.022; (**b**) ε = 0.044; (**c**) ε = 0.066; (**d**) ε = 0.088; (**e**) ε = 0.110; (**f**) ε = 0.132; (**g**) ε = 0.154; (**h**) ε = 0.176.

Figure 21 presents the damage evolution characteristics of the material subjected to tensile loading along the z-axis direction under 2b/w = 0.4. This color representation clearly demonstrates a layered damage propagation pattern along the specimen height: damage initially appears at z = 0.30 × 10^−4^ m, subsequently expands to z = 2.0 × 10^−4^ m and then to z = 2.3 × 10^−4^ m, and ultimately permeates the entire structure. This finding is also consistent with the phenomenon that increasing crack size leads to premature material fracture.

### 4.2. Macroscopic Model

#### 4.2.1. Crack Size

**Figure 22:**
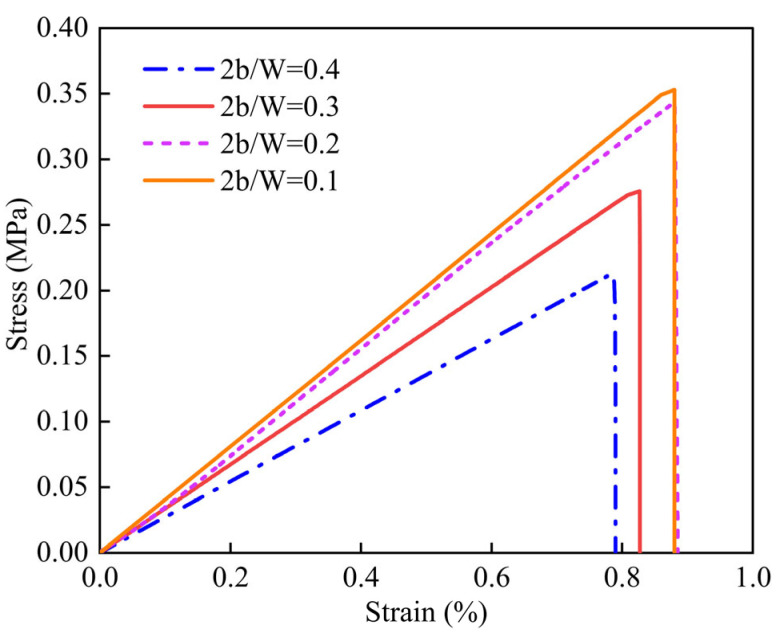
Stress–strain curves of CFITs with different crack size under tensile loading.

Figure 22 shows stress–strain curves of CFITs with different crack sizes under tensile loading. The crack size (2b/W) are 0.1, 0.2, 0.3 and 0.4, respectively. It can be seen that the stress required for material fracture exhibits a progressively decreasing trend with increasing fracture width. Crack sizes below 0.3 result in a negligibly altered stress–strain curve, indicating a minimal effect on the CFIT’s mechanical properties. In contrast, when the crack size is no less than 0.3, the stress required for fracture decreases markedly, reflecting a significant reduction in load-bearing capacity caused by larger crack size. This observed trend is consistent with the predictions from the mesoscopic model.

**Figure 23:**
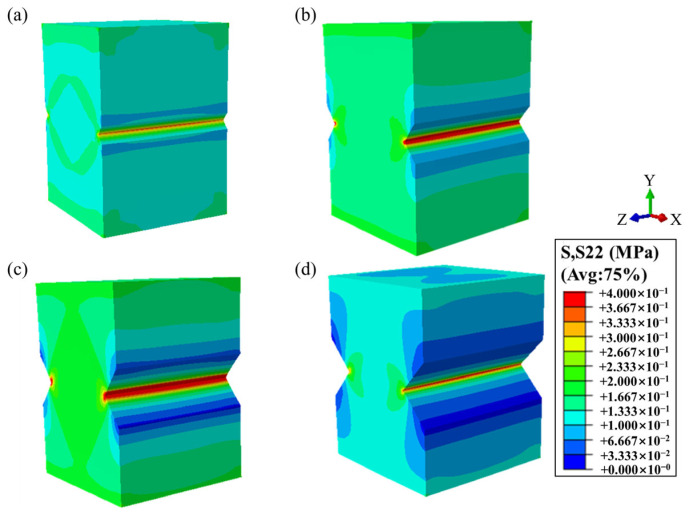
Stress contour map of CFITs with different crack size under tensile loading. (**a**) 2b/W = 0.4; (**b**) 2b/W = 0.3; (**c**) 2b/W = 0.2; (**d**) 2b/W = 0.1.

Figure 23 illustrates the stress contour map of CFITs with different crack size under tensile loading. In CFITs featuring various crack sizes, the corresponding fracture cloud maps are consistently characterized by symmetric distributions, which conform to the stress field features characteristic of mode I fracture as described in linear elastic fracture mechanics. A notable stress concentration arises at the flaw tip, from which stress waves propagate uniformly outward in a radial manner. The propagation distance of these stress waves scales positively with crack size, indicating that a larger flaw gives rise to a more extensive range of stress wave propagation.

#### 4.2.2. Prefabricated Defect

**Figure 24:**
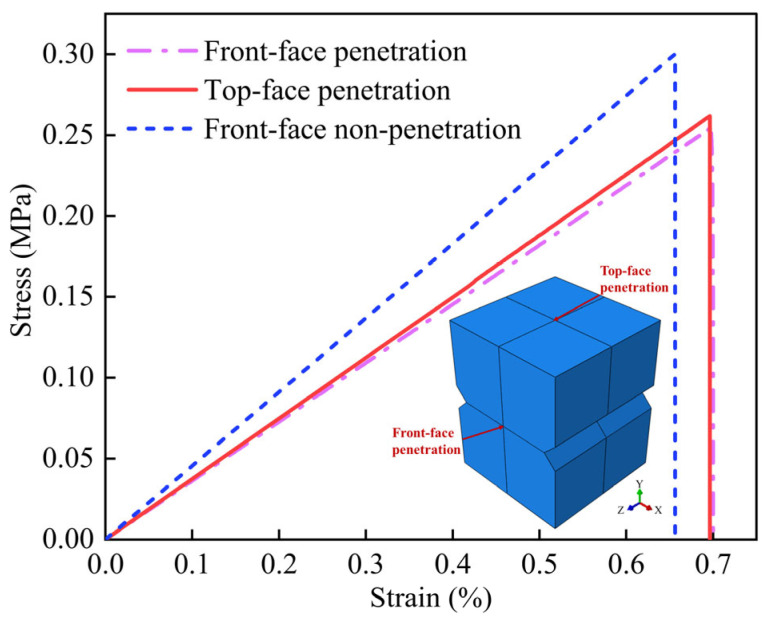
Stress–strain curves of CFITs with different prefabricated defects under tensile loading.

To investigate the influence of prefabricated defects on the CFITs’ fracture property, three defect configurations were examined in this study: front-face penetration, top-face penetration, and front-face non-penetration. The penetration defects are defined as cylindrical through-holes with a bottom diameter of 1 mm, whereas the non-penetration defect is defined as a cylindrical blind hole with a bottom diameter of 1 mm and a depth of 2 mm. All crack sizes (2b/W) are 0.2. Figure 24 shows the stress–strain curves of CFITs with different prefabricated defects under tensile loading. As shown in Figure 24, the stress–strain curves for specimens with front-face and top-face penetration defects nearly overlap, both showing a maximum fracture stress of approximately 0.25 MPa. This indicates that the penetration direction has no significant effect on fracture property, and its weakening impact on the CFIT’s fracture strength is limited. In contrast, more pronounced brittle fracture behavior is observed in the specimen with a front-face non-penetration defect. This defect configuration yields a maximum fracture stress of 0.31 MPa, which is approximately 24% higher than that of the penetration defect specimens. This illustrates that while the non-penetration defect increases brittleness, it also enables the CFIT to sustain a higher fracture load.

**Figure 25:**
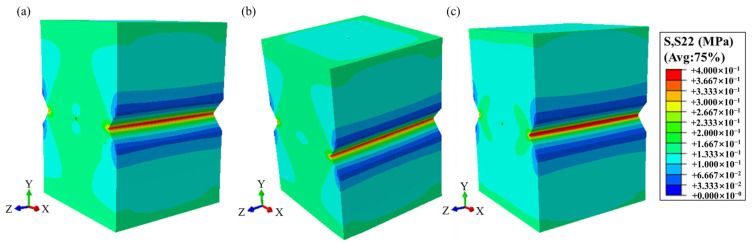
Stress contour map of CFITs with different prefabricated defects under tensile loading, (**a**) front-face penetration; (**b**) top-face penetration; (**c**) front-face non-penetration.

Figure 25 shows the stress contour map of CFITs with different prefabricated defects under tensile loading. It can be seen that the stress contour maps for front-face and top-face penetration defects are highly consistent, with only minor local fluctuations near the penetration opening. This suggests that the penetration direction has a limited effect on the internal stress field. The stress wave is generated at the flaw tip, propagates radially outward, and can extend beyond the geometric constraint of the penetration defect. Meanwhile, a pronounced stress concentration persists at the flaw tip, serving as the primary driver for crack initiation and propagation. In contrast, the non-penetration defect specimen exhibits a distinctly different stress response. Stress concentration remains highly localized at the flaw tip, from which the stress wave also propagates radially. Except at the crack tip, the stress field over the non-penetration cross-section is generally uniform, and no appreciable stress concentration is evident. However, due to the finite-depth blind hole, overall fracture initiates prematurely in the shallow region before the stress wave reaches the defect bottom, preventing adequate reflection and reloading at the hole bottom. This alters the crack propagation path and significantly affects the component’s load-bearing capacity and failure mode. Thus, the stress wave does not fully traverse the defect region, and the defect bottom exerts limited influence on fracture behavior. This observation provides a rationalization for the simultaneous increase in fracture strength and brittleness associated with the non-penetration defect.

## 5. Discussions

Both mesoscopic and macroscopic numerical results demonstrate that fibrous porous material such as CFIT is insensitive to cracking, which is in good agreement with Ref. [28]. Increasing the fiber length from 0.05 mm to 0.1~0.15 mm enhances fracture toughness, owing to an optimal length range that suppresses defect generation from long fiber clustering. Moderately reducing the fiber diameter can control the specific surface area and improve processing uniformity, but excessive reduction compromises the intrinsic fiber strength and thus degrades the overall fracture property. The above results provide theoretical guidance for toughening CFIT. Meanwhile, macroscopic modeling of prefabricated defect locations can provide theoretical guidance for hole positioning in CFIT during practical applications. While the mesoscopic model employed provides fracture toughness values, its size limitation restricts the results to a qualitative trend assessment, precluding quantification. For future work, coupling the mesoscopic and macroscopic models would enable the prediction of the mechanical response of CFIT at the actual scale.

## 6. Conclusions

This study developed a mesoscopic fracture model based on a refined three-dimensional random network structure to investigate CFIT fracture behavior under varying crack size, porosity, fiber length, diameter, and orientation angle. The fracture property of CFIT is insensitive to crack size below the critical threshold (2b/W < 0.3). Meanwhile, a negative correlation is observed between porosity and CFIT’s strength as well as its stiffness. A fiber length of 0.1~0.15 mm enhances toughness, while moderate refinement of fibers contributes to the enhancement of fracture toughness in CFIT. As the fiber orientation angle increases, fracture property declines slowly and then plateaus. In summary, this study reveals the regulatory effect of mesoscale parameters on CFIT’s fracture toughness, identifies the critical crack size threshold, and provides theoretical guidance for the investigation of mesoscopic fracture behavior under tensile loading.

A macroscopic FE model of CFIT was established, and the numerically simulated fracture toughness agreed well with experimental results. The crack size threshold for fracture properties agrees with that of the mesoscale model, and the stress wave propagation range widens as the crack size increases. Front-face and top-face penetration defects show similar effects on fracture behavior. The non-penetration defect achieves approximately 24% higher fracture stress than the penetration defect. This is attributed to greater material continuity and reduced local stress concentration, which delays unstable crack propagation. However, this strength gain is accompanied by increased brittleness. These findings provide a theoretical basis for understanding the fracture behavior of CFIT. Current studies on the tensile fracture behavior of CFIT are largely confined to single factor analyses, with multi-factor coupling effects remaining unexplored. Moreover, the present work does not account for temperature variations. Consequently, future efforts should systematically examine the influence of temperature on fracture behavior, particularly the mechanisms by which temperature affects fracture modes and damage evolution.

## Data Availability

The original contributions presented in this study are included in the article/Supplementary Materials. Further inquiries can be directed to the corresponding author.

## Supplementary Materials

The following supporting information can be downloaded at: https://www.mdpi.com/article/10.3390/ma19153246/s1, Figure S1: Mesh convergence result of ceramic fiber insulation tile; Table S1: Mesh region division of CFIT.
