# Supplementary material for: Fracture Behavior and Mechanism of Ceramic Fiber Insulation Tiles: An Analysis Based on Experiment and Numerical Simulation
Source: Materials (Basel). 2026 Jul 31;19(15):3246. doi: 10.3390/ma19153246 (PMC13467948; doi:10.3390/ma19153246)
Supplement: Supplementary file 1 [file materials-19-03246-s001.zip › materials-4454202-supplementary.pdf]

Supplementary Information

Fracture Behavior and Mechanism of Ceramic Fiber Insulation Tiles: An Analysis Based on Experiment and Numerical Simulation

Yiming Wang [a], Hong Ye [b], Xiaoliang Ma [a], Xuefeng Mu [b], Kaili Yin [a], Meng Cui [a], Baojun Zhao [a],Yuanpeng Fang [b], Yesheng Zhong [a,*], Liping Shi [a] and Xiaodong He [a]

[a] National key laboratory of science and technology on advanced composites in special environments, Harbin Institute of Technology, Harbin 150080, China.

[b] Chengdu Aircraft Design and Research Institute, Aviation Industry Corporation of China, Chengdu 610091, China

* Correspondence: zhongyesheng@hit.edu.cn (Y. Zhong)

Supplementary Note 1: Mesh convergence of ceramic fiber insulation tile

To systematically evaluate the discretization error and verify the mesh convergence of the numerical simulation, four mesh refinement strategies were designed for the ceramic fiber insulation tile (CFIT). The mesh sizes are specified in millimeters (mm), with distinct settings for the non-fracture region and the fracture region. The four configurations are as follows:

Table S1. Mesh region division of CFIT

Number	Non-fracture region size	Fracture region size
1	2.5	1.414
2	1.5	1.414
3	2.0	2.828
4	2.0	0.707

Figure S1 shows the mesh convergence result of CFIT. For the non-fracture region, the computational errors obtained with mesh sizes of 2.5 and 1.5 are 15.6% and 18.8%,

respectively, both exceeding that associated with a mesh size of 2.0. When the mesh size in the fracture region is set to 2.828, the solution fails to converge; however, refining the mesh to 0.707 mm does achieve convergence, but the associated error is as high as 21.9%, far exceeding that obtained with a mesh size of 1.414 mm. These results indicate that the optimal mesh configuration is a combination of 2.0 in the non-fracture region and 1.414 in the fracture region.

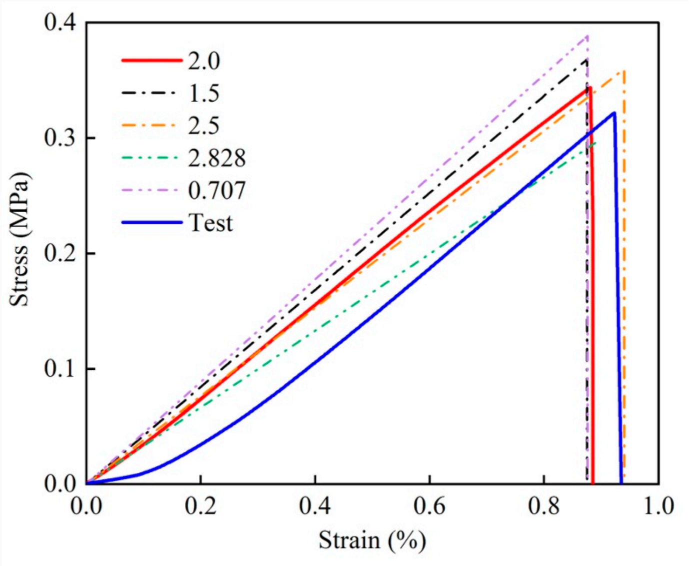

Figure S1. Mesh convergence result of ceramic fiber insulation tile
